# Specific phenotypic, genomic, and fitness evolutionary trajectories toward streptomycin resistance induced by pesticide co-stressors in *Escherichia coli*

**DOI:** 10.1038/s43705-021-00041-z

**Published:** 2021-08-18

**Authors:** Yue Xing, Xiaoxi Kang, Siwei Zhang, Yujie Men

**Affiliations:** 1 Department of Chemical and Environmental Engineering, University of California, Riverside, CA USA; 2 Department of Civil and Environmental Engineering, University of Illinois at Urbana–Champaign, Urbana, IL USA

**Keywords:** Bacterial genetics, Population genetics, Environmental sciences

## Abstract

To explore how co-occurring non-antibiotic environmental stressors affect evolutionary trajectories toward antibiotic resistance, we exposed susceptible *Escherichia coli* K-12 populations to environmentally relevant levels of pesticides and streptomycin for 500 generations. The coexposure substantially changed the phenotypic, genotypic, and fitness evolutionary trajectories, resulting in much stronger streptomycin resistance (>15-fold increase) of the populations. Antibiotic target modification mutations in *rpsL* and *rsmG*, which emerged and dominated at late stages of evolution, conferred the strong resistance even with less than 1% abundance, while the off-target mutations in *nuoG*, *nuoL*, *glnE*, and *yaiW* dominated at early stages only led to mild resistance (2.5–6-fold increase). Moreover, the strongly resistant mutants exhibited lower fitness costs even without the selective pressure and had lower minimal selection concentrations than the mildly resistant ones. Removal of the selective pressure did not reverse the strong resistance of coexposed populations at a later evolutionary stage. The findings suggest higher risks of the selection and propagation of strong antibiotic resistance in environments potentially impacted by antibiotics and pesticides.

## Introduction

Antibiotic resistance poses a major threat to public health worldwide. It has been estimated that 700 000 people died every year due to antibiotic-resistant bacterial infections during 2014 and 2016, globally [1]. This number is even predicted to reach as many as 10 million by the year 2050 if no actions are taken [1]. In addition to the increase of antibiotic-resistant pathogens in clinical settings, antibiotic-resistant bacteria and resistance genes are frequently detected in natural and engineered environments, such as surface water, soil, wastewater, and sludge [2–5]. Resistant bacteria developed in those environments may re-enter the water cycle and food chain, potentially imposing health risks. Thus, it is urgent to control the emergence and spread of antibiotic resistance in those environmental hotspots.

The resistance of the microorganisms could either be obtained by *de novo* mutations through evolution or mediated by horizontal transfer of resistance genes. The evolution of antibiotic resistance is driven by selective pressures [6]. Antibiotics are the long-standing focus to study selective pressures. Antibiotics, both at lethal levels and below minimal inhibitory concentrations (sub-MIC), are able to facilitate resistance evolution, including the selection of *de novo* resistant mutants and the selection of preexisting resistant mutants over a long-term selection [7–12]. The two selection levels of antibiotics can induce specific genetic mutations, which render different resistance mechanisms to microbial populations [6, 11, 12]. With lethal levels of antibiotics, cells either die or survive, depending on the spontaneous acquisition of resistance-specific mutations, which in general confer high levels of resistance. In contrast, antibiotics at sub-MIC levels do not kill cells and tend to favor more diverse mutations, the combination of which may also cause strong phenotypic resistance [11].

The resistance developed at sub-MIC levels raises more environmental concerns because in many environments, such as surface water, wastewater, biosolids, agricultural soils, and surface runoffs [13–19], antibiotics occur at sub-MIC levels. However, in most of those environments, antibiotic residues do not exist alone. Other organic contaminants, such as non-antibiotic drugs, personal care products, and pesticides, often coexist with antibiotics [19–21]. Nevertheless, co-selection by two or more stressors remains poorly understood. Our recent study reveals a synergistic effect of pesticides on the development of resistant mutants under the selection of sub-MIC ampicillin [22]. As a result, the evolution of antibiotic resistance could have been underestimated in environments with the presence of both antibiotics and non-antibiotic chemicals. This study also raised more fundamental questions on how co-stressors would shape a bacterial population during a long-term evolution and how mutational dynamics would affect the phenotypes in terms of antibiotic resistance and growth fitness.

The process of antibiotic resistance evolution could be divided into the emergence of resistant mutants, the proliferation, and maintenance of resistant subgroups in a bacterial population. Previous studies that identified stressors selecting antibiotic resistance mainly focus on resistant mutants and mutations developed at the endpoint of the evolution [11, 12, 22–25]. These studies facilitated the understanding of the interplay between stressors and the most beneficial mutations. However, this type of study fails to demonstrate the sustainability of the resistant lineages in a population, the dynamics of genetic adaptation, and the associations of resistance evolution with genome and fitness evolution. All of these factors are of great importance to understand the spread and persistence of resistant bacteria in their receiving environments, as well as to predict and intervene in the evolution of antibiotic resistance.

In this study, we aimed to fill the knowledge gap and correlate the evolutionary trajectories in *Escherichia coli* populations coexposed to antibiotics and non-antibiotic co-stressors among the three interactive aspects: the resistance phenotype, genetic mutations, and growth fitness of mutants. We conducted laboratory evolution experiments by exposing a susceptible *E. coli* K-12 strain to sub-MIC streptomycin (Strep) and non-antibiotic organic chemicals (i.e., pesticides or non-antibiotic pharmaceuticals) for 500 generations. We chose Strep as the exposed antibiotic because Strep is one of the human antibiotics that have been widely used in plant agriculture to combat bacterial diseases like the citrus greening disease [26], highly likely co-occurring with pesticides and non-antibiotic drugs. It belongs to aminoglycoside antibiotics. Mechanisms of Strep resistance include target modification (altering protein binding site by mutations of the target genes, like *rpsL*) [11, 12] and decrease of Strep uptake [11]. Instead of the endpoint evaluation of resistance development, we focused on the trajectories of Strep resistance, genomic, and fitness evolution. We investigated the associations of evolutionary trajectories of Strep resistance with the trajectories of genetic mutations and mutant growth fitness in the coexposed populations. Stimulated development of strong Strep resistance by the non-antibiotic co-stressors, pesticides but not pharmaceuticals, was demonstrated. Novel mutations leading to Strep resistance were identified, which expands our fundamental knowledge of antibiotic resistance mechanisms developed under environmentally relevant exposure conditions. Moreover, relative fitnesses of the identified resistant mutants in the coexposed populations were determined, which could be used to predict the proliferation and spread of certain resistant mutants once they are transported into a different environment.

## Materials and methods

### The selection of pesticides and pharmaceuticals

Given that organic contaminants often occur in the environment in mixtures, in this study, we decided to use a mixture of pesticides or non-antibiotic pharmaceuticals (two representative groups of environmental organic contaminants) as the non-antibiotic selective pressure in the experimental evolution, although they may not necessarily all occur in the same environment. We arbitrarily selected the pesticides and pharmaceuticals (Table 1), which were frequently detected in the environment. Since streptomycin is used as both drugs and pesticides, it likely co-occurs with pesticides and other non-antibiotic pharmaceuticals in various natural and engineered environments, such as agricultural runoffs and wastewater treatment plants. We used the average concentrations reported in the literature the representative concentrations in the environment (e.g., for pesticides: 0.1 – 4.8 µg/L each and ~20 µg/L in total).

**Table 1:** Environmental concentrations of selected pesticides and pharmaceuticals.

Pesticides	Category	Mode of action	Occurring environment^a^	Conc. used (μg/L)
2,4-D	Herbicide	Synthetic plant hormone	**Urban run-off** [48], wastewater [49]	0.2
Mecoprop	Herbicide	Synthetic plant hormone	**Urban run-off** [48], wastewater [50]	2
Benomyl	Fungicide	Inhibits cell division	**Surface water** [51]	0.2
Metolachlor	Herbicide	Inhibits cell division	**Wastewater** [52], surface water [53]	0.4
Thiabendazole	Fungicide	Inhibits cell division	**Wastewater** [19, 54], surface water [55]	0.2
Carbaryl	Insecticide	Acetylchlolin-esterase inhibitor; nervous system disruptor	**Surface water** [56], wastewater [49]	4.8
Carbofuran	Insecticide	Acetylchlolin-esterase inhibitor; nervous system disruptor	**Subsurface and surface water** [57], wastewater [52]	0.38
Chlorpyrifos	Pesticide	Acetylchlolin-esterase inhibitor; nervous system disruptor	**Lake** [58], wastewater [59]	0.4
Diazinon	Insecticide	Acetylchlolin-esterase inhibitor; nervous system disruptor	**Wastewater** [52], surface water [60]	0.3
Fipronil	Insecticide	Acetylchlolin-esterase inhibitor; nervous system disruptor	**Urban surface water** [61], wastewater [62]	0.2
Imidacloprid	Insecticide	Acetylchlolin-esterase inhibitor; nervous system disruptor	**Subsurface and surface water** [57], wastewater [62]	0.4
Propiconazole	Fungicide	Inhibits sterol synthesis and damage membrane permeability	**Wastewater** [63], lake [64]	1
Imazalil	Fungicide	Inhibits sterol synthesis and damage membrane ﻿permeabilit﻿y	**River** [65], wastewater [52]	0.4
Clotrimazole	Fungicide	Inhibits sterol synthesis and damage membrane ﻿permeabilit﻿y	**Wastewater** [64], surface water [66]	0.1
Irgarol	Biocide	Inhibits photosynthesis	**Coastal water** [67], wastewater and surface water [68]	0.2
Linuron	Herbicide	Inhibits photosynthesis	**Rivers** [69], wastewater [70]	2
Diuron	Herbicide	Inhibits photosynthesis	**Urban run-off** [48], wastewater [49]	1
Atrazine	Herbicide	Inhibits photosynthesis	**Subsurface and surface water** [71], wastewater [72]	0.5
Terbuthylazine	Herbicide	Inhibits photosynthesis	**Subsurface and surface water** [57], wastewater [49]	0.65
Terbutryn	Herbicide	Inhibits photosynthesis	**Rivers** [73], wastewater [68]	0.5
Tebuconazole	Fungicide	Inhibits spore spread	**Wastewater** [74], lake [64]	0.5
DEET	Biocide	Interferes with neurons and receptors	**Wastewater influent** [20], surface water [50]	3
Metaldehyde	Pesticide	Produces mucus	**Surface water** [75], wastewater [76]	0.5
			**Total**	**19.83**
**Pharmaceuticals**	**Uses/Mode of action**		**Occurring environment**	**Conc. used (μg/L)**
Acetaminophen	Antipyretic and analgesic drug		**Wastewater** [20], surface water [46]	5
Ibuprofen	Nonsteroidal anti-inflammatory drug		**Wastewater** [77], surface water [46]	2
Carbamazepine	Anticonvulsant		**Wastewater** [20], surface water [46]	2
Gabapentin	Anticonvulsant		**Wastewater** [20], surface water [78]	5
Atenolol	Beta-blocker		**Wastewater** [79], surface water [46]	4
Caffeine	Psychoactive drug, nervous system stimulant		**Wastewater** [20], surface water [46]	5
Amantadine	Treat Parkinson’s disease and influenza A virus infection		**Wastewater** [80]	1
Fluconazole	Antifungal (binding to fungal cytochrome P450)		**Wastewater** [81], surface water [46]	0.5
Flucytosine	Antifungal (interfering with fungal RNA and protein) synthesis		**Wastewater** [20]	1
Ranitidine	H_2_ blocker		**Wastewater** [20], surface water [46]	2
O-desmethylvenlafaxine	Active metabolite of venlafaxine, an antidepressant drug		**Wastewater** [20], surface water [46]	1
			**Total**	**28.5**

^a^Concentration used in the study was detected in the environment indicated in bold.

### Bacterial strains, growth, and evolutionary experiments

The bacterial strain used in this study was Gram-negative *Escherichia coli* K-12 strain (ATCC. 10798), which has been widely used as the susceptible and negative control in many relevant studies [27–31]. The growth medium for all evolutionary experiments was Luria-Bertani (LB) broth. First, the stock *E. coli* cells were revived and then streaked on an LB agar plate. After 20-hour incubation, one single colony was picked and inoculated into LB broth, which was regarded as the ancestor (G0), and used as the inoculum for the evolutionary experiments.

The selective pressures included a combination of Strep and pesticides, Strep and pharmaceuticals, pesticides only, pharmaceuticals only, and Strep only. The concentrations of Strep included 1/5 MIC_0_ (the MIC of G0, 8 mg/L), which represented a low-level antibiotic selection. Three pesticide/pharmaceutical exposure levels were included, i.e., 1×, 10×, and 100× environmental concentrations [denoted (1/5Strep,1 P), (1/5Strep,10 P), and (1/5Strep,100 P) for pesticides]. The control groups without chemical exposure were also set up using eight independent populations. Moreover, to take into account the potential dose-effect caused by the additional amount of pesticide co-stressor besides the primary stressor (1/5MIC_0_ Strep), we included a condition of (1/2Strep,0), where the concentration of Strep (4 mg/L) was comparable to the total concentration (3.6 mg/L) of (1/5Strep,100 P). Cell growth under different exposure conditions was measured by optical density at 600 nm (OD_600_) (See Supplementary Methods in the Supplementary Information for details).

**Fig. 1:**
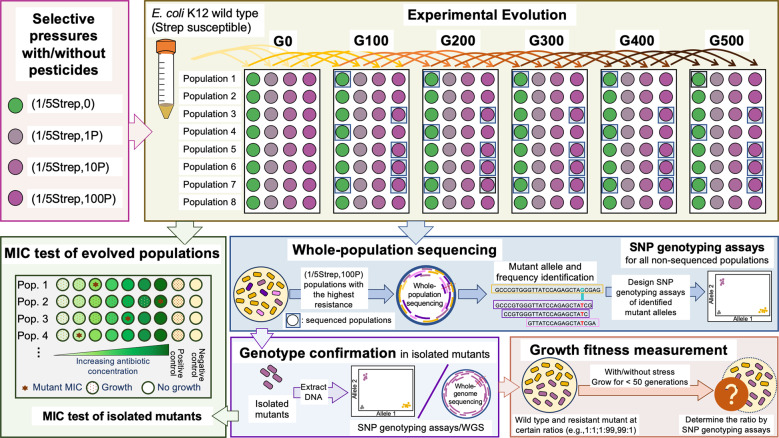
Illustration of the experimental design. A total of eight parallel populations of *E. coli* K-12 under each exposure condition were serially passaged every 24 h (dilution factor = 1:500, ~9 generations) into fresh LB medium containing Strep and/or pesticides at the same exposure levels for 500 generations. The exposure conditions include 1/5 MIC_0_ of Strep only, denoted “(1/5Strep,0)”; 1/5 MIC_0_ of Strep and the pesticide mixture: (1/5Strep,1 P), (1/5Strep,10 P), and (1/5Strep,100 P); pesticides only: (0,1 P), (0,10 P), and (0,100 P); and the no exposure control: (0, 0). “P” represents environmental concentrations of the pesticides as listed in Table 1. Populations after every 100 generations (highlighted in blue boxes) were subject to the whole-population sequencing. All other populations after every 100 generations, as well as select isolated mutants were subject to single nucleotide polymorphisms (SNP) genotyping assays for genotype confirmation.

Evolutionary experiments were performed as described in our previous study [22]. We serially passaged eight replicate populations of each condition in LB media containing certain concentrations of pesticides/pharmaceuticals and Strep (Fig. 1). During each transfer, the cell culture was first diluted ten times, and then a volume of 4 µL diluted inoculum was inoculated into each well (500× dilution), making the total volume of 200 µL. The plate for each transfer was incubated at 30 °C in a 150-rpm shaker in the dark every 24 h. The exposure was conducted for about 500 generations (~55 passages). The cultures after every 100 generations were preserved by adding 100 µL of 50% glycerol and stored at −80 °C.

### MIC test of evolved populations

Every 100 generations, the evolved populations were subject to MIC tests, which determine phenotypic resistance levels of the populations. The cell culture was diluted with 0.9% NaCl solution to an OD_600_ of 0.1, which was regarded as the standard solution. Then 0.5 μL of the standard solution was added into fresh LB medium containing Strep with a series of concentrations. A growth control without the antibiotic and a negative control without bacterial inoculum was set up in the meanwhile. Cell cultures were incubated at 37 °C for 20 h, and then the OD_600_ was measured. The MIC was determined as the concentration that inhibited 90% of growth based on the OD_600_ measurement [9, 32]. We then performed the Wilcoxon rank-sum test to analyze the difference of population MICs after 500 generations under coexposure conditions and single exposure to Strep (*p*-value <0.05, *N* = 8).

### DNA extraction, whole-population sequencing, and single nucleotide polymorphisms (SNP) calling

To study the differences in mutational dynamics caused by the addition of pesticides, we selected three replicate populations from (1/5Strep,0) and four replicate populations from (1/5Strep,100P), (0, 100P), and (0, 0). The archived populations at every 100 generations for the (1/5Strep, 0) and (1/5Strep, 100P) conditions (only the 500-generation populations were sequenced for the (0, 100P) and (0, 0) conditions) were cultivated overnight in LB medium, and cell pellets were collected by centrifugation. Genomic DNA (gDNA) was extracted using the DNeasy Blood and Tissue Kit (Qiagen) according to the manufacturer’s instructions. The gDNA concentration and quality were determined on a Qubit 4 Fluorometer (Thermo Fisher Scientific, Wilmington, DE). The gDNA was then subjected to Illumina NovaSeq 150-bp paired-end sequencing carried out by Roy J. Carver Biotechnology Center at the University of Illinois. The average coverage was 13.3 M paired-reads per sample. A dynamic sequence trimming was done by SolexaQA software [33] with a minimum quality score of 30 and a minimum sequence length of 50 bp. All samples were aligned against the *E. coli* K-12 MG1655 genome available at NCBI GenBank (NC_000913.3) using the Bowtie 2 toolkit [34]. SAMtools was used to format and reformat the intermediate alignment files [35]. SNPs and insertions, and deletions (INDELs) were identified and annotated with software BCFtools [36] and SnpEff [37]. The valid mutant alleles in the sequenced populations were those with (i) amino-acid-sequence change, (ii) not found in the ancestor G0, (iii) >8-read coverage, and (iv) >10% mutant allele frequency at the mutation positions.

To examine the similarity of mutational spectra leading to strong resistance between (1/5Strep,100 P) and (1/2Strep,0), we included three populations from (1/2Strep,0), which developed similar levels of strong resistance after 200 generations for whole-population sequencing with the same analysis procedure described above.

### Genotype confirmation of non-sequenced populations and isolated resistant mutants

The generality of the identified mutations was examined by SNP genotyping assays (See Supplementary Methods for details) for the non-sequenced populations, including the evolved populations coexposed to (1/5Strep,0), (1/5Strep,1P), (1/5Strep,10P), and (1/5Strep,100P) at every 100 generations, as well as the evolved populations of (0,100P) and (1/2Strep,0) at the end of experimental evolution.

We then isolated resistant mutants in the evolved populations onto selective LB agar plates (1× MIC_0_ Strep), including (1/5Strep,100 P)-3 at G300, (1/5Strep,100 P)-5 at G400, (1/5Strep,100P)-6 at G300, (1/5Strep,100P)-3 at G500, and (1/2Strep,0)-1 at G200. Three resistant mutant colonies were picked up from each population and subjected to gDNA extraction. The gDNA was tested for the presence/absence of the six mutant alleles. As no successful SNP assays could be designed for the *yaiW* and *rsmG* mutations that were also likely responsible for the resistance phenotype, the genotypes of the resistant mutants isolated from (1/5Strep,100 P) populations and those from (1/2Strep,0) populations were further confirmed by whole-genome sequencing following the same procedure as whole-population sequencing described above, except for the mutation frequency greater than 50%.

### MIC tests of isolated mutants and mock populations

To examine the correlation between mutant genotypes and their corresponding resistance levels, we determined the MICs of the isolated mutants with different genotypes (three mutants for each genotype). We then statistically analyzed the difference of MICs among the isolated mutants using the Wilcoxon rank-sum test (*p*-value < 0.05, *N* = 3).

To determine the correlation between the population MICs and fractions of strongly resistant mutants, we constructed mock populations by growing the mutant and wild-type cells at various ratios. The tested fractions of the resistant mutant in the mock populations included 0, 10^-5^, 10^–4^, 10^–3^, 10^–2^, 10%, 20%, 30%, 40%, 50%, 60%, 70%, 80%, 90%, 99%, 1. These populations were then subject to the same MIC tests, as described above.

### Growth fitness measurement by competition tests

The competition tests were first carried out in pairs of wild type vs. the mildly resistant mutants, wild type vs. the strongly resistant mutant, and mildly resistant mutant vs. the strongly resistant mutant. The resistant clones were those with confirmed genotypes isolated from the evolved populations. Mock populations were constructed by inoculating two types of cells at a ratio of 1:99 and 99:1, separately. Six parallel populations were inoculated for 50 generations under the selection of (1/5Strep,100 P) or no selective pressure. The mock populations were all grown in the same conditions as used in the experimental evolution (i.e., nutrient-rich LB medium, 30 °C, shaking at 150 rpm in dark, and transferring every 24 h). The fractions of the two types of cells in the population at the end of cultivation were then determined by comparing the allelic discrimination plots from SNP genotyping assays to those of standard mixtures (Fig. S1). The minimal selective concentration (MSC) of resistant mutants was determined (See the Supplementary Methods).

We then compared the relative fitness of resistant mutants from (1/5Strep,100 P) with those from Strep only selections, including (1/5Strep,0)-1 at G500, (1/5Strep,100 P)-4 at G500, (1/5Strep,100 P)-7 at G500, and (1/2Strep,0) at G200. The ratio of competition pairs is approximately 1:1. Each competition pair was grown in the same conditions as described above. The fraction of (1/5Strep,100 P) mutants after 18 generations was determined by SNP genotyping assays. The relative fitness was calculated according to the selection coefficient of cell A/cell B: ln[R(t)/R(0)]/t, as previously described [38], where R is the ratio of cell A to cell B, t is growth generation. The selection coefficient >0 stands for higher fitness of cell A to cell B. We also used this system to evaluate the individual contribution of pesticides and 1/5 MIC_0_ to the selection of preexisting mutants.

## Results

### Trajectories of phenotypic resistance to Strep in *E. coli* populations with and without pesticide co-stressors

**Fig. 2:**
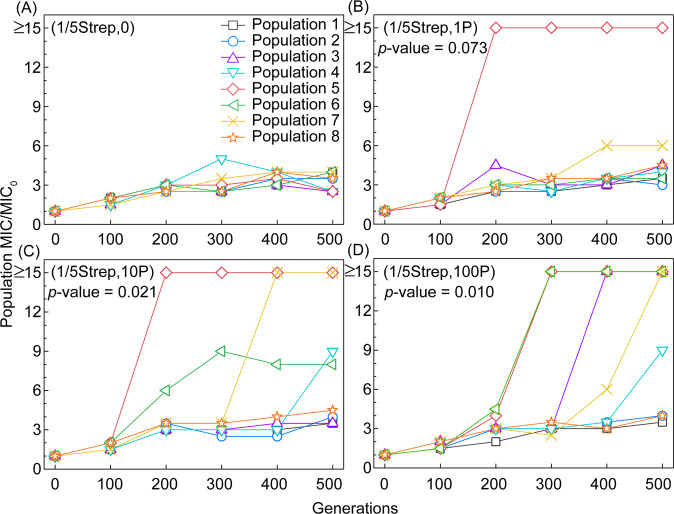
Evolution of Strep-resistance phenotype of *E. coli* populations under different exposure conditions. Population MICs to Strep over 500 generations under different exposure conditions (**A**): (1/5Strep,0), (**B**): (1/5Strep,1 P), (**C**): (1/5Strep,10 P), (**D**): (1/5Strep,100 P) (*p* values are from the Wilcoxon rank-sum test between the MICs of the coexposed populations and the Strep-only exposure control after 500 generations).

The exposures to Strep at 1/5 MIC_0_ (i.e., 1/5Strep) and/or the pesticides at the highest level (i.e., 100P) (Fig. 1) did not significantly inhibit cell growth, with only 1.7–2.9% decrease in the maximum growth rate (Table S1). However, the coexposure to pesticides under 1/5Strep selective pressure substantially altered the evolutionary trajectories of the *E. coli* populations toward high-level resistance. The resistance trajectories of (1/5Strep,0) over 500 generations revealed similar trends among the eight parallel populations, which only acquired low-level Strep resistance with 2.5–4× increase in MIC (Fig. 2A). In contrast, when exposed to even 1P pesticides together with 1/5Strep, one out of the eight populations acquired much stronger resistance (≥15× increase in MIC) (Fig. 2B). The other seven (1/5Strep,1P) populations showed similar evolutionary trajectories of Strep resistance as those of the (1/5Strep,0) populations. We observed a dose-effect of pesticide co-stressors on the stimulated evolution toward Strep resistance. More coexposed populations became strongly Strep-resistant as the pesticide level increased, i.e., 2/8 and 4/8 populations exhibited strong resistance (i.e., ≥15× increase in MIC) for the (1/5Strep,10P) and (1/5Strep,100P) exposures, respectively (Fig. 2C, D). The strong Strep resistance can emerge from 200 generations after the coexposure (1/5Strep,1P), where the pesticide concentration (20 µg/L) was much lower than Strep (1.6 mg/L) (Fig. 2B). The MICs of populations coexposed to (1/5Strep,10P) and (15Strep,100P) were significantly different from those of populations exposed to only Strep after 500 generations, according to the Wilcoxon rank-sum test (*p* < 0.05). It indicates that despite the random emergence of resistance, the observed increase in resistance in the coexposed populations was mainly driven by the pesticide co-stressor. In contrast, the emergence of one lineage with higher resistance after the exposure to (1/5Strep, 1P) was not statistically significant (with a cutoff *p* value of 0.05) and could be due to stochastic effect.

The replicate populations in the absence of chemical exposure did not obtain increased resistance levels (Table S2), which suggests that the growth conditions, such as the liquid medium and temperature, and passage manner after every 24 h, in general, did not drive antibiotic resistance evolution. Pesticide-only exposures caused ~1.5× increase in MIC in a portion of the eight populations (Table S2). The combined effect of pesticides and Strep in selecting for resistance is much greater than the sum of individual effects of pesticides or Strep, which suggests a synergistic effect. Moreover, the coexposure to 1/5Strep and select pharmaceuticals (Table 1) did not exhibit a significant difference in MIC increase from that acquired under the exposure to 1/5Strep only (Table S3). Hence, we demonstrated the specificity of pesticides as co-stressors in significantly promoting the evolutionary trajectories toward high-level Strep resistance.

### Distinctive mutational dynamics along the resistance evolutionary path in *E. coli* populations coexposed to Strep and pesticides

To couple phenotypic resistance trajectories with genetic adaptation, we carried out whole-population sequencing to identify the mutational dynamics under different selective pressures with/without pesticides. Since more populations evolved under (1/5Strep,100P) have high-level resistance compared to the other two pesticide exposure conditions, we selected the four independent populations exposed to (1/5Strep,100P) (i.e., populations 3, 5, 6, and 7), which acquired the highest resistance (≥15× increase in MIC) (Fig. 2D), for the whole-population sequencing every 100 generations (i.e., G0, G100, G200, G300, G400, and G500). Three parallel populations from (1/5Strep,0) (i.e., populations 1, 4, and 7) (Fig. 2A) were also sequenced as a comparison. We also sequenced four replicate populations exposed to (0, 100P) and (0, 0) after 500 generations to examine the effect of pesticide-only exposure and LB medium on the genetic evolution. Valid mutant alleles (one mutant allele represents one specific mutation in a gene) in the evolved populations were called out (Table S4) using standardized variant calling procedures [22] and based on two criteria: (i) mutations leading to amino-acid-sequence change, including non-synonymous SNPs and INDELs, and (ii) mutant allele frequency in the population was larger than 10% at least at one time point. To have a comprehensive profile of mutational dynamics, we retrieved the frequencies of valid mutant alleles along the evolutionary path from G100 to G500.

**Fig. 3:**
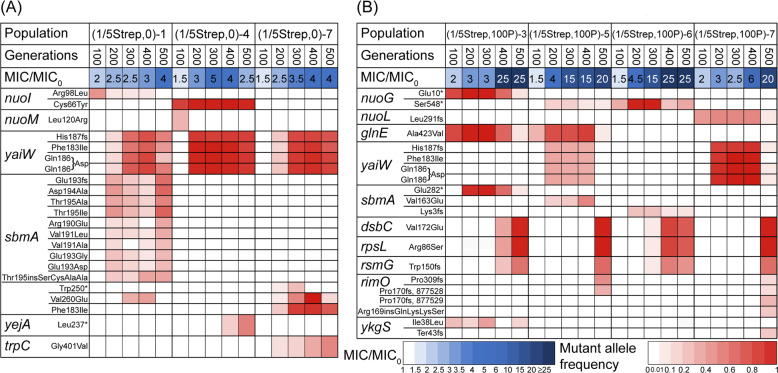
All mutated genes, the frequency of mutant alleles in the sequenced populations from G100 to G500, and the corresponding MICs. **A**: three parallel populations exposed to (1/5Strep,0); **B**: four parallel populations exposed to (1/5Strep,100 P).

In the (0, 0) and (0, 100P) populations, very few missense mutations were detected (one in the (0, 0) populations and three in the (0, 100P) populations) (Table S4 & S5), suggesting that the LB medium and the pesticide-only exposure did not stimulate genetic mutation, and it is consistent with no increase in the Strep-resistance phenotype (Table S2). In comparison, we observed multiple mutations and distinctive differences in mutational dynamics between Strep-exposed populations with and without pesticide co-stressors, which corresponded to the different phenotypic Strep resistance trajectories. All the mutations were different from those detected in the (0, 0) and (0, 100P) conditions. At an early stage in the evolved populations coexposed to (1/5Strep,100P), mutant alleles of certain genes emerged at G100 or G200, including *nuoG* (stop-gained mutations: Glu10* and Ser548*, * represents a stop codon), *nuoL* (Leu297fs, “fs” represents frameshifting), *glnE* (Ala423Val)*, yaiW* (various SNPs), and *sbmA* (various SNPs), whose abundances continued to increase and became dominant after G200 or G300 (Fig. 3B). In line with that, there was a slight increase in population MICs (2–6×) (Fig. 3B). The populations exposed to (1/5Strep,0), which also developed mild resistance (1.5–5× increase in population MICs), acquired mutant alleles in the same or similar genes, such as *yaiW*, and *sbmA* (Fig. 3A). For example, the same *yaiW* mutant alleles as in (1/5Strep,100 P)-7 were detected in all three sequenced (1/5Strep,0) populations.

**Fig. 4:**
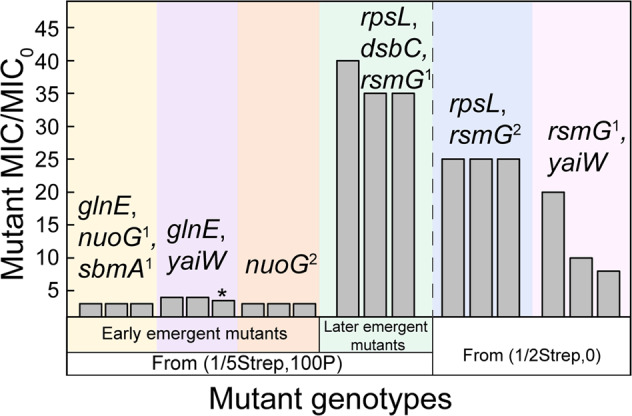
MICs of Strep-resistant mutants with different genotypes. For each genotype, three mutants were investigated; mutants containing *glnE* (Ala423Val), *nuoG*^*1*^ (Glu10*), and *sbmA*^*1*^ (Glu282*) mutations were isolated from population (1/5Strep,100P)-3 at G300; mutants containing the same *glnE* mutation and *yaiW* mutations (Phe183Ile, Gln186Asp, His187fs) were isolated from (1/5Strep,100P)-5 at G400 [Note: the third mutant marked with “*” in the figure also had the *aidB* mutation (Pro159Gln)]; mutants containing *nuoG*^*2*^ (Ser548*) mutation were isolated from (1/5Strep,100P)-6 at G300; mutants with *rpsL* (Arg86Ser), *dsbC* (Val172Glu), and *rsmG*^*1*^ (Trp150fs) mutations were isolated from (1/5Strep,100P)-3 at G500; and mutants with the same *rpsL* and *rsmG*^*2*^ (Ser15insGly) mutations were isolated from (1/2Strep,0)-1 at G200).

The above mutations occurred in genes encoding proteins that are not targeted by Strep, known as off-target mutations. Those genes are involved in: (i) electron transport (i.e., *nuoG* and *nuoL*), (ii) membrane permeability and transport (i.e., *yaiW* and *sbmA*), (iii) metabolism (i.e., *glnE*) and (iv) phage (i.e., *ykgS*). We then isolated resistant clones carrying these mutations. The off-target mutations in *nuoG*, *glnE*, *sbmA*, and *yaiW* were all associated with a mild (3–4×) increase in Strep resistance (Fig. 4, Fig. S2). Strep belongs to aminoglycoside antibiotics, which are cationic molecules. Its uptake depends on electron transport through quinones and a high membrane potential [39]. The *nuoG* mutations have been reported to reduce the uptake of Strep [11], leading to Strep resistance. The *nuoG* mutations identified in our study were stop-gaining mutations, which may lead to loss of function. In addition, *yaiW* and *sbmA*, two closely located genes in the *E. coli* genome, encode outer membrane proteins related to antimicrobial peptide transportation [40]. Mutations in *sbmA* have been detected previously in resistant *E. coli* mutants developed under gradually increasing selective pressures of aminoglycosides [25]. The mutations in *sbmA* may result in reduced membrane permeability [41], thus reducing the uptake of Strep. The *yaiW* mutations have not been identified in previous studies. Given the similar gene function of *yaiW* and *sbmA*, *yaiW* mutations likely had the same resistance mechanism as *sbmA* mutations. So far, there have not been any reports on links between Strep resistance and *glnE* mutations. The *glnE* mutation could be a resistance mutation but also could be a compensatory mutation, which could reduce the fitness cost of other mutations in the mutants.

As some of those mutations were all detected with high frequencies (80–100%), they seemed to accumulate in the same mutants in the evolved population. The accumulation of mutations was observed in both (1/5Strep,0) populations and early-stage (1/5Strep,100P) populations, for example, *nuoI*, *yaiW*, and *sbmA* in populations (1/5Strep,0)-4 and seven, as well as *nuoG*, *glnE*, and *sbmA* in (1/5Strep,100 P)-3 at G200 (Fig. 3). The combination of mutations failed to significantly increase in MIC of those populations. For example, the population dominated by a combination of *nuoG*, *sbmA*, and *glnE* mutations only showed 3× MIC_0_. This result was corroborated by the MICs of resistant clones carrying *nuoG*, *sbmA*, and *glnE* mutations, which were also 3× MIC_0_ (Fig. 4, Fig. S2).

Although populations exposed to (1/5Strep,0) and (1/5Strep,100P) shared the same or similar trajectories of phenotypic and genotypic resistance before G300, the (1/5Strep,100 P) populations exhibited a distinct succession of mutant alleles afterward. The mutations in genes *rpsL* (Arg86Ser), *rsmG* (Trp150fs), and *dsbC* (Val172Glu) occurred in all replicated (1/5Strep,100P) populations after G300. As the new mutations appeared, Strep resistance of those populations increased substantially (15 – 25×) (Fig. 3B). In the meanwhile, the early-emergent mutant alleles started to fade or completely disappeared in the populations (Fig. 3B). In contrast, the (1/5Strep,0) populations did not develop any mutations in *rpsL*, *rsmG*, or *dsbC* during the studied evolution period (Fig. 3A).

The *rpsL* gene encodes a Strep target protein (30S ribosomal protein S12). Mutations in *rpsL* have been frequently reported in resistant clones under strong Strep selection (e.g., a lethal dose of Strep) [11, 12, 42]. For example, the same *rpsL* mutation (Arg86Ser) was reported in an *E*. *coli* K-12 strain exposed to increasing Strep concentrations (close to and above MIC_0_) along the evolutionary path [12]. *rpsL* mutations may cause structure alteration of the target protein and reduction of Strep binding affinity. In our study, the same genetic mutation in *rpsL* was also induced under 1/2MIC_0_ Strep selection (Table S4), implying that the strength in selecting de novo mutants of (1/5Strep,100P) is as strong as 1/2MIC_0_ Strep. Resistant clones with the *rpsL* mutation were isolated from populations under both selection conditions, which conferred strong phenotypic resistance (25–40×) (Fig. 4). More importantly, the same *rpsL* mutation and strong resistance were also developed under the coexposure to (1/5Strep,10P), where the pesticide level was 10× lower (Table S6). It suggests that the synergistic effect of 1/5 MIC_0_ Strep and pesticide co-stressors was not simply because the addition of pesticides increased the total stressor level. Instead, pesticides might play a different role from Strep in driving the evolution towards higher resistance under the coexposure.

The *rsmG* (*gidB*) gene encodes a methyltransferase involved in the methylation of the 16S rRNA. The loss of RsmG activity could dwindle the binding of Strep to the 30S subunit like the *rpsL* mutations, leading to Strep resistance [43, 44]. The loss-of-function mutations in *rsmG* have been identified in previous studies [11, 43], which conferred mild to strong Strep resistance. The *rsmG* mutation in (1/5Strep,100P) populations was a 25 bp frameshift deletion, which likely compromised the enzyme activity, thus resulting in Strep resistance. The same deletion mutation was also detected in mutants from the (1/2Strep,0) population (Table S4), which showed an 8–20× increase in MIC (Fig. 4). The other identified mutations in that population were only in *yaiW*. As the same *yaiW* mutations in the (1/5Strep,0) populations only resulted in mild resistance, the strong Strep resistance (i.e., 20× MIC_0_) in the (1/2Strep,0) population (Table S7) was more likely caused by the 25 bp frameshift deletion in *rsmG*.

The *dsbC* mutation co-occurred with the *rpsL* mutation at the same frequencies in the (1/5Strep,100P) populations after G300 (Fig. 3B), as well as in the other coexposed populations that acquired strong resistance (Table S5), suggesting that the two mutations were developed in the same genomes. Furthermore, among the randomly picked ten isolates from coexposed populations, all isolates with the *rpsL* mutation also carried the *dsbC* mutation (Table S8), demonstrating that *rpsL* and *dsbC* were coevolved. More interestingly, the co-selection of *rpsL* and *dsbC* mutations was not observed in populations exposed to 1/2MIC_0_ Strep (Table S4). Mutations in *dsbC* were not linked to antibiotic resistance previously. The function of this mutation could either contribute to the resistance or reduce the fitness cost of costly resistance mutations. The isolated resistant clones with *rpsL*, *dsbC*, and *rsmG* mutations in (1/5Strep,100P) populations exhibited a significantly (*p* < 0.01) stronger resistance than that of resistant clones isolated from (1/2Strep,0), which also carried mutations in *rpsL* and *rsmG* (Fig. 4). Given the similar levels of resistance conferred by the two loss-of-function mutations of *rsmG*, the *dsbC* mutation was likely involved in the resistance elevation.

**Fig. 5:**
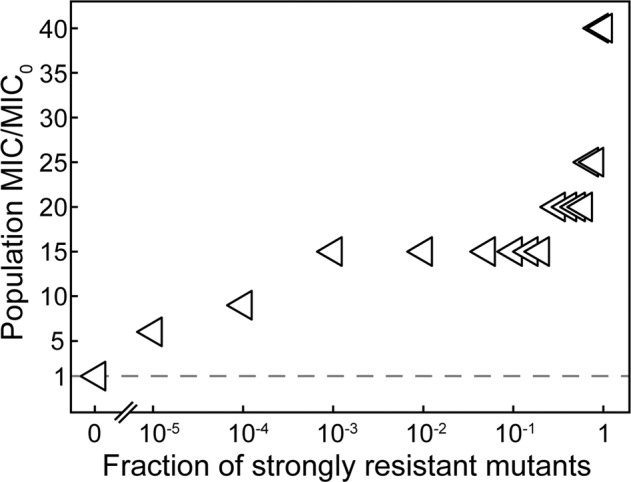
The effect of relative abundance of the strongly resistant mutant on the Strep resistance in mock populations. MICs of mock populations containing the wild type and various fractions of the strongly resistant mutant carrying the *rpsL*, *dsbC*, and *rsmG* mutations.

A significant increase in the phenotypic resistance showed up right after the first emergence of the *rpsL* + *dsbC* + *rsmG* mutant in the (1/5Strep,100P) populations. We used a titration experiment to determine the lowest fraction of strongly resistant mutation that could cause a change in the phenotypic resistance of the population. We constructed mock populations containing the strongly resistant *rpsL* + *dsbC* + *rsmG* mutant and the wild type at different ratios. We observed that even when the fraction of the strongly resistant mutant was lowered to 10^−3^, the population MIC still increased significantly to 15× MIC_0_ (Fig. 5). A fraction as low as 10^–5^ still caused a five-fold increase in MIC of the population. This result could explain the high-level resistance obtained in the (1/5Strep,100 P)-5 population at G300 and G400 (Fig. 3B), where mutations conferring strong resistance were not identified. The strongly resistant mutants might have already arisen in the populations, which led to the substantial increase in the population MIC, but the mutation frequency could be too low (i.e., <1/sequencing depth ~1/800) to be detected by the whole-population sequencing.

### Fitness evolutionary trajectories in *E. coli* populations under the coexposure

**Fig. 6:**
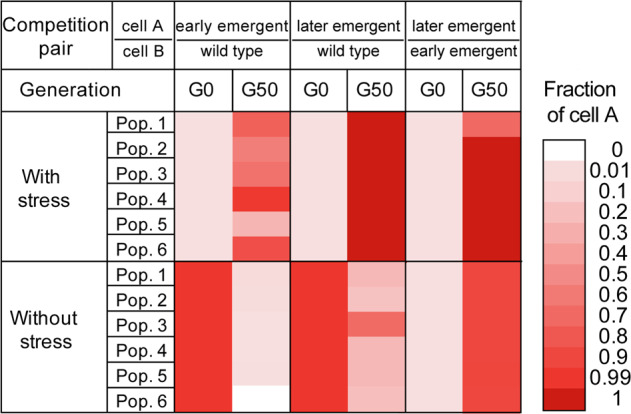
Growth fitness of the emerged Strep-resistant mutants under the coexposure. Growth competition between the wild type, the early emergent mutant with mild Strep resistance (carrying *nuoG, glnE, and sbmA* mutations), and the late emergent mutant with strong Strep resistance (carrying *rpsL*, *dsbC*, and *rsmG* mutations) in LB medium with and without the selective pressure (1/5Strep,100 P). Six parallel populations containing cell A and B were performed; two initial fractions of cell A, i.e., 1% and 99%, were included.

The relative fitness of resistant mutants in a population determines their evolutionary directions with or without selective pressure. For example, if the resistant mutants had higher fitness than the wild type in the presence of the selective pressure, the wild type would be outcompeted and toward extinction in the population, leading to the persistence of phenotypic resistance. According to the succession pattern in the coexposed populations, the late emergent mutants with strong Strep resistance seem to have the highest fitness, followed by the early emergent mutants, whereas the wild type has the lowest fitness under the selective pressure of (1/5Strep,100P). This was corroborated by competition tests between the wild type, the mildly resistant mutant (with *nuoG*, *glnE*, and *sbmA* mutations), and the strongly resistant mutant (with *rpsL*, *dsbC*, and *rsmG* mutations). Expectedly, starting at 1% in the population, both the mildly and strongly resistant mutants outcompeted the wild-type cells under the selective pressure (1/5Strep,100P) after 50 generations (Fig. 6), indicating higher growth fitness of those resistant mutants than the wild type. Additionally, the strongly resistant mutant outcompeted the mildly resistant mutant in constructed cocultures coexposed to (1/5Strep,100P), consistent with the succession observed in the actual coexposed populations. The same result (Fig. S3) was observed when the strongly resistant mutant was competing with the other two early emergent mutants, which were isolated from the coexposed populations and conferred mild resistance. Furthermore, we examined the individual roles of Strep (i.e., 1/5MIC_0_) and pesticides (i.e., 100P) in selecting preexisting resistant mutants over wild type. The results reveal that exposure to pesticides did not change the relative fitness of resistant mutants and wild type compared to the no-stress condition, and the presence of Strep is the key factor favoring the growth of preexisting resistant mutants over the wild type (Table S9). We then estimated the minimal selective concentrations (MSC) of Strep for these resistant mutants by competing them with wild-type cells. There was variation in the MSC of different mutants; the lowest MSC corresponded to 1/40 MIC_0_ (i.e., 200 μg/L) for the late emergent mutant with strong resistance, while the highest MSC was 1/10 MIC_0_ for the mutant with a combination of *nuoG*, *glnE*, and *sbmA* mutations (Table S10). These results imply that enrichment of the preexisting resistant mutants is possible at Strep concentrations significantly below the MICs of wild-type strains, and with a lower MSC the strongly resistant mutant could even be more favorably selected over the mildly resistant mutants in a population.

Resistant mutants usually have fitness costs and are likely to become extinct in competition with wild-type cells in the absence of selective pressures, leading to the reversal of antibiotic resistance evolution. To test the reversibility of the evolution direction after the removal of the selective pressure, we conducted competition tests starting with 99% of the mildly or strongly resistant mutants and 1% of wild-type cells in LB broth without any selective pressure. As expected, when competing against the wild type without the selective pressure, both mildly and strongly resistant mutants lost their growth advantages and were outcompeted after 50 generations (Fig. 6, Fig. S3). This also suggests that acquired mutations were not involved in the co-adaptation of the *E. coli* to the specific culture conditions, such as liquid media, temperature, and passage manner. Interestingly, in competitions between mildly and strongly resistant mutants without the selective pressure, the strongly resistant mutant still outcompeted the mildly resistant mutant even when the relative abundance of the strongly resistant mutant was as low as 1% (Fig. 6, Fig. S3). It demonstrated a higher fitness of the strongly resistant mutant than the mildly resistant mutant regardless of the selective pressure, indicating that the evolution direction may not be reversed by simply removing the selective pressure in a population where the two mutant types coexist. We further demonstrated this by growing (1/5Strep,100P)-5 (G500) population in LB medium without the selective pressure. After 50 generations, the fraction of strongly resistant mutant with *rpsL* + *dsbC* + *rsmG* mutations remained at ~100% in the population (Table S11). We also explored the reversibility of resistance evolution in populations from the selection of 1/2MIC_0_ Strep. The fraction of the strongly resistant mutant dropped from nearly 100% to 5% in (1/2Strep,0)-1 after 50 generations (Table S11). These results indicate that the genetic backgrounds of the populations that evolved under both exposure conditions are quite different, which affected the reversibility of the evolution.

**Fig. 7:**
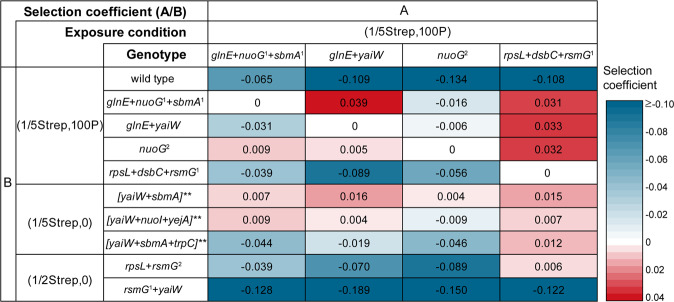
Selection coefficients of cells with different genotypes isolated from (1/5Strep,100 P) populations and mutants from Strep only selection conditions. Mutations: *glnE* (Ala423Val), *nuoG*^*1*^ (Glu10*), *sbmA*^*1*^ (Glu282*), *yaiW* (Phe183Ile, Gln186Asp, His187fs), *nuoG*^*2*^ (Ser548*), *rpsL* (Arg86Ser), *dsbC* (Val172Glu), *rsmG*^*1*^ (Trp150fs), and *rsmG*^*2*^ (Ser15insGly); []** indicates the suspect genotypes of the mutant based on the whole-population sequencing results.

We then compared the relative fitness of resistant mutants from (1/5Strep,100P) populations with those from Strep-only conditions. The more positive the selection coefficient (A/B) is, the higher fitness cell A has over cell B, and vice versa. From the results, we could draw two conclusions. First, the mutant with the highest fitness came from the (1/2Strep,0) condition at G200 with *rsmG* + *yaiW* mutations, followed by the mutant from the (1/5Strep,100P) condition at G500 with *rpsL* + *dsbC* + *rsmG* mutations. The mutant with the most significant fitness defect was the resistant clone from (1/5Strep,0)-1 at G500 (Fig. 7). Usually, it has been known that the stronger the selection strength, the lower the fitness of the obtained resistant mutants [6, 24]. However, our results strongly fail to support this perspective. On the contrary, we observed mutants with a higher fitness from the stronger selection conditions, whereas the most defective mutant evolved from the weakest selection condition. This implies that the fitness of resistant mutants might be dependent on their characteristics with regard to specific mutations, not the strength of selection. Second, the relative fitness of *rpsL* + *dsbC* + *rsmG* mutant is slightly higher than *rpsL* + *rsmG* mutant, suggesting the role of *dsbC* mutation in reducing the fitness cost caused by resistance mutations (e.g., *rpsL* mutation).

## Discussion

Selective pressures are of great importance to understand the evolution of antibiotic resistance in the environment. Previous studies have demonstrated that sub-MIC antibiotics can serve as selective pressures and facilitate the evolution of antibiotic resistance [45]. However, very little is understood about how non-antibiotic co-stressors could affect the antibiotic resistance selection and the phenotypic, genomic, and fitness evolutionary trajectories. Here, we focused on the evolution of antibiotic resistance in microbial populations under the coexposure to sub-MIC (i.e., 1/5MIC_0_) Strep and the co-stressor, pesticides. We first showed that the exposure to pesticides, in addition to sub-MIC Strep, led to the selection of strong resistance, which could not be driven by the Strep-only exposure. Next, we coupled the evolutionary trajectories of the phenotypic resistance with the evolutionary trajectories of genotypes and growth fitness. We observed the succession of dominant mutants in the coexposed populations from the off-target mutations to the target-modification mutations during the evolution. This displacement pattern led to the transition from mild to strong phenotypic resistance. Compared to the mildly resistant mutants, the strongly resistant mutants developed under the coexposure exhibited higher growth fitness with and without the selective pressure, as well as a lower MSC, which could favor the proliferation and sustain the dominance of strongly resistant mutants under environmentally relevant conditions.

There are several implications of these results. First, the role of pesticide co-stressors in promoting the selection of strong antibiotic resistance would be of greater concern, which has been largely overlooked. The concentration of pesticides to exhibit the synergistic effect could be as low as 20 µg/L in total. It is a typical level in many agricultural and wastewater-related environments, where antibiotics could coexist at low levels [21, 46, 47]. Thus, such environmental conditions may select for *de novo* mutants with much stronger resistance than those selected by the same level of antibiotics alone. Pharmaceutical co-stressors examined in this study did not perform in the same way as pesticides, suggesting one pesticide or certain groups of pesticides could uniquely promote antibiotic resistance selection as co-stressors. The synergistic effect seems not due to accelerated mutagenesis by the pesticide cocktail to the *E. coli* populations, as the pesticide-only exposure did not stimulate genetic mutation. We also demonstrated that pesticides are not involved in the selection of preexisting resistant mutants, which is solely dependent on the antibiotic. The MSC of Strep for the obtained mutants could be as low as 1/40 MIC_0_. This result is consistent with the previous study, which suggests the selection of preexisting resistant mutants could be much lower than inhibitory concentrations [8]. Thus, future studies may focus on individual pesticides to pinpoint those that can cause the synergistic effect, as well as the mechanisms of the pesticide co-stressors driving the evolution toward higher-level resistance. Since our previous study demonstrated the synergistic effect of pesticides and another antibiotic (ampicillin) on the selection of de novo resistant mutants [22], we are inclined to advocate that the synergistic effect is not limited to one antibiotic. Nonetheless, it is worth more comprehensive studies on the synergistic effect of pesticides with a series of antibiotics from different categories.

Second, our results showed various mutations leading to Strep resistance, including some novel resistance mechanisms. Target-modification mutations like *rpsL* mutations and *rsmG* mutations have been reported to cause strong resistance to Strep [11, 12, 44]. However, off-target mutations conferring resistance are less known. In this study, by studying the evolutionary trajectories of genomic evolution, we have identified novel off-target mutations conferring mild Strep resistance. For example, the mutations in *yaiW* and *sbmA* genes, which encode peptide antibiotic transporters on cell membranes, might reduce membrane permeability [41], and thus lower the uptake of Strep. Notably, the accumulation of off-target mutations failed to lead to strong resistance. This result disagrees with the previous study, where the combination of five mutations led to a significantly high level of resistance, although individual mutations did not confer strong resistance [11]. It might be due to the difference in acquired mutation spectra, exposure length (900 vs. 500 generations), and tested bacterial species (*S. enterica* vs. *E. coli*) between the two studies. Moreover, our results implied that the *dsbC* mutation, which was coevolved with *rpsL* under the coexposure, could elevate Strep resistance and lower the fitness cost caused by other resistance mutations. These results need to be followed with mechanistic studies to determine the precise functional roles of the individual mutations by constructing site-specific mutants. Next, the prevalence of these mutations evolved from laboratory systems needs to be examined in the environments potentially impacted by antibiotics and pesticides. If the resistance mutations could be identified in real environments, they could serve as biomarkers indicative of antibiotic resistance.

Third, effective mitigation strategies against the development and propagation of strong antibiotic resistance should be more carefully and comprehensively made considering selective pressures, genetic background, and subgroup fitness comparison in microbial populations under varying environmental conditions. The evolutionary trajectories in this study highlighted the succession from genotypes conferring mild resistance to those conferring strong resistance in coexposed populations, which was not observed in populations exposed to sub-MIC Strep only. Removal of stressors is one strategy to control the development and/or proliferation of strongly resistant mutants, hence reducing antibiotic resistance in a population. However, it might not work effectively for the populations that have developed strong resistance from a transitional phase dominated by mildly resistant mutants with lower fitness. Moreover, since some resistant mutants likely have an MSC much lower than MIC, high removal efficiencies of antibiotics would be needed to avoid the selection of antibiotic-resistant mutants. Collectively, the presence of antibiotics and other co-stressors in specific environments makes it more challenging to control antibiotic resistance. In this case, pesticides and antibiotics need to be effectively removed at an early stage of exposure or even before they enter the receiving environment, for example, enhancing their removal efficiencies in wastewater treatment plants.

## Supplementary information

Supplementary Information

Supplementary TableS4

## Data Availability

All whole-population/genome sequencing data have been deposited in the NCBI SRA database under Accession No. PRJNA605244.
