# Supplementary material for: Specific phenotypic, genomic, and fitness evolutionary trajectories toward streptomycin resistance induced by pesticide co-stressors in Escherichia coli
Source: ISME Commun. 2021 Aug 18;1:39. doi: 10.1038/s43705-021-00041-z (PMC9723568; doi:10.1038/s43705-021-00041-z)
Supplement: Supplementary file 1 — Supplementary Information [file 43705_2021_41_MOESM1_ESM.pdf]

Supplementary Information for

Specific phenotypic, genomic, and fitness evolutionary trajectories toward streptomycin resistance induced by pesticide co-stressors in *Escherichia coli*

Supplementary Methods

Measuring growth rate under different conditions

Growth rates were measured at 37 °C in non-selective LB broth using Synergy 2 Multi-Mode Microplate Reader (BioTek Instruments). Each population exposed to (0,0), (1/5Strep,0), (1/5Strep,100P), and (0,100P) had 10 biological replicates on a 96-well plate. The inoculum was the culture grown overnight from 10 µL of 2× diluted archived population in 2 mL LB broth. The 96-well plate was incubated in the Microplate Reader for 24 h with continuous shaking at 150 rpm in the dark, and the optical density at 600 nm (OD_{600}) was measured every 15 min. The max growth rates and lag time were calculated by the software Gen 5.

SNP genotyping assays

We designed PCR-based SNP genotyping assays via Custom TaqMan SNP Genotyping Assays (Thermo Fisher Scientific) targeting six parallel mutant alleles: *nuoG*^{1} (Glu10*), *nuoG*^{2} (Ser548*), *glnE* (Ala423Val), *sbmA*^{1} (Glu282*), *rpsL* (Arg86Ser), and *dsbC* (Val172Glu). The genotyping reactions were carried out in a total volume of 5 µL (2.75 µL master mix + customized genotyping assay and 2.25 µL diluted DNA sample of 3 ng/µL) according to the manufacturer's instructions. The assays were performed in 96-well plates on a real-time PCR instrument QuantStudio 3 (Thermo Fisher Scientific), following the recommended thermal cycling conditions. Thermo Fisher Cloud "Genotyping" application was used to generate allele calls.

The SNP genotyping assay was used to confirm genotypes of isolated mutants and also adapted to estimate (i.e., semi-quantify) the mutant allele frequencies in non-sequenced coexposed populations along the evolutionary path and in constructed cocultures for the competition tests. The quantification standards were prepared by mixing gDNA of cell A with gDNA of cell B at different ratios, resulting in the fraction of the mutant alleles at 1%, 5%, 10%, 15%, 20%, 30%, 40%, 50%, 60%, 70%, 80%, 90%, 95%, and 99%. The fraction of mutant alleles in the tested populations was estimated by comparing the allelic discrimination plot to those of the standard mixtures (Fig. S1).

Determination of minimal selective concentrations (MSC)

The MSC was estimated as the streptomycin concentration where the resistant mutant has an equal growth rate with the wild type. We set up competition tests between a resistant mutant and the wild type (1:1) with Strep addition ranging from 0 to 1/2 MIC_0 (0, 1/100, 1/80, 1/40, 1/20, 1/10, 1/8, 1/5, 1/2). The MSC was determined as the lowest concentration of Strep, at which the fraction of the resistant mutant did not decrease after the competition test.

Table S1 The max V and lag time of *E. coli* ancestor (G0) populations grown under different selection pressure

**(0,0)**		**(1/5Strep,0)**		**(1/5Strep,100P)**		**(0,100P)**	
**Max V**	**Lag time**	**Max V**	**Lag time**	**Max V**	**Lag time**	**Max V**	**Lag time**
5.864	2:15:15	5.446	2:13:16	5.708	2:17:42	5.651	2:07:23
5.179	2:06:50	5.759	2:11:40	5.477	2:10:30	5.486	2:06:06
6.073	2:04:07	5.099	2:04:36	5.727	2:08:20	5.632	1:59:17
5.265	1:59:36	5.599	2:05:09	5.568	2:11:37	5.055	1:58:20
5.653	2:00:22	5.877	2:09:30	5.116	1:58:05	5.479	1:56:45
5.601	2:02:51	5.372	1:59:08	4.739	1:58:54	5.35	2:03:17
5.89	2:03:46	5.341	2:03:41	5.08	1:59:16	5.362	2:01:08
5.582	1:58:52	5.705	2:05:17	5.645	2:01:44	5.356	1:56:52
6.115	2:09:45	5.868	2:07:11	5.692	2:02:39	5.758	2:01:08
5.362	1:56:16	5.531	2:11:02	5.255	2:02:33	5.763	1:57:14
Average							
5.658	2:03:46	5.56	2:07:03	5.4	2:05:08	5.489	2:00:45

Table S2 Strep resistance developed in *E. coli* populations exposed to only pesticides after 500 generations

Population	MIC (MIC_0 = 8 mg/L)			
	(0,0)	(0,1P)	(0,10P)	(0,100P)
Rep-1	1× MIC_0	1× MIC_0	1× MIC_0	1× MIC_0
Rep-2	1× MIC_0	1× MIC_0	1× MIC_0	1× MIC_0
Rep-3	1× MIC_0	1.5× MIC_0	1× MIC_0	1.5× MIC_0
Rep-4	1× MIC_0	1.5× MIC_0	1.5× MIC_0	1.5× MIC_0
Rep-5	1× MIC_0	1.5× MIC_0	1.5× MIC_0	1.5× MIC_0
Rep-6	1× MIC_0	1.5× MIC_0	1.5× MIC_0	1.5× MIC_0
Rep-7	1× MIC_0	1.5× MIC_0	1.5× MIC_0	1.5× MIC_0
Rep-8	1.5× MIC_0	1.5× MIC_0	1.5× MIC_0	1.5× MIC_0

Table S3 Strep resistance developed in *E. coli* populations exposed to Strep at 1/5MIC_0 and pharmaceuticals (Ph) after 500 generations

**Population**	**MIC (MIC_0 = 8 mg/L)**			
	**(1/5Strep,0)**	**(1/5Strep,1Ph)**	**(1/5Strep,10Ph)**	**(1/5Strep,100Ph)**
Rep-1	4× MIC_0	4× MIC_0	4× MIC_0	4× MIC_0
Rep-2	4× MIC_0	4× MIC_0	4× MIC_0	4× MIC_0
Rep-3	4× MIC_0	6× MIC_0	4× MIC_0	4× MIC_0
Rep-4	4× MIC_0	4× MIC_0	4× MIC_0	4× MIC_0
Rep-5	6× MIC_0	4× MIC_0	4× MIC_0	6× MIC_0
Rep-6	4× MIC_0	4× MIC_0	6× MIC_0	6× MIC_0
Rep-7	4× MIC_0	4× MIC_0	4× MIC_0	4× MIC_0
Rep-8	6× MIC_0	6× MIC_0	4× MIC_0	4× MIC_0

Table S5 Number of mutations in populations at 500G compared to G0

Population	Number of mutations	Number of valid mutations[a]
(0,0)-1	16	1
(0,0)-2	20	2
(0,0)-3	25	1
(0,0)-4	21	1
(0,100P)-1	21	3
(0,100P)-2	19	1
(0,100P)-3	23	1
(0,100P)-4	20	2

[a]valid mutations refer to mutations leading to amino-acid-sequence change.

Table S6 SNP detection of mutations identified by sequencing in the non-sequenced populations

Populations	Generation	MIC[a] (×MIC_0)	*nuoG* (E10*)	*nuoG* (S548*)	*glnE* (A423V)	*sbmA* (E282*)	*rpsL* (R86S)	*dsbC* (V172E)
(1/5Strep,0)-2	G100	2	×[b]	×	×	×	×	×
	G200	2.5	×	×	×	×	×	×
	G300	2.5	×	×	×	×	×	×
	G400	3.5	×	×	×	×	×	×
	G500	3.5	×	×	×	×	×	×
(1/5Strep,0)-3	G100	1.5	×	×	×	×	×	×
	G200	3	×	×	×	×	×	×
	G300	2.5	×	×	×	×	×	×
	G400	3	×	×	×	×	×	×
	G500	2.5	×	×	×	×	×	×
(1/5Strep,0)-5	G100	2	×	×	×	×	×	×
	G200	3	×	×	×	×	×	×
	G300	3	×	×	×	×	×	×
	G400	3.5	×	×	×	×	×	×
	G500	2.5	×	×	×	×	×	×
(1/5Strep,0)-6	G100	2	×	×	×	×	×	×
	G200	3	×	×	×	×	×	×
	G300	2.5	×	×	×	×	×	×
	G400	3	×	×	×	×	×	×
	G500	4	×	×	×	×	×	×
(1/5Strep,0)-8	G100	2	×	×	×	×	×	×
	G200	2.5	×	×	×	×	×	×
	G300	2.5	×	×	×	×	×	×
	G400	4	×	×	×	×	×	×
	G500	3.5	×	×	×	×	×	×
(1/5Strep,1P)-1	G100	1.5	×	×	1–5%[c]	×	×	×
	G200	2.5	×	×	×	×	×	×
	G300	2.5	×	×	×	×	×	×
	G400	3	×	×	×	×	×	×
	G500	3.5	×	×	×	×	×	×
(1/5Strep,1P)-2	G100	2	×	×	×	×	×	×
	G200	2.5	×	×	×	×	×	×
	G300	2.5	×	×	×	×	×	×
	G400	3.5	×	×	×	×	×	×
	G500	3	×	×	×	×	×	×
(1/5Strep,1P)-3	G100	1.5	×	×	×	×	×	×
	G200	4.5	×	×	×	×	×	×
	G300	3	×	×	×	×	×	×
	G400	3	×	×	×	×	×	×
	G500	4.5	×	×	×	×	×	×
(1/5Strep,1P)-4	G100	2	×	×	×	×	×	×
	G200	3	×	×	×	×	×	×
	G300	2.5	×	×	×	×	×	×
	G400	3.5	×	×	×	×	×	×
	G500	4	×	×	×	×	×	×
(1/5Strep,1P)-5	G100	1.5	×	×	×	×	×	×
	G200	25	×	×	×	×	×	×
	G300	25	×	×	×	×	×	×
	G400	25	×	×	×	×	×	×
	G500	25	×	×	×	×	×	×

Lineage	Generation	MIC (×MIC_0)	*nuoG* (E10*)	*nuoG* (S548*)	*glnE* (A423V)	*sbmA* (E282*)	*rpsL* (R86S)	*dsbC* (V172E)
(1/5Strep,1P)-6	G100	2	×	×	×	×	×	×
	G200	3	×	×	×	×	×	×
	G300	3	×	1%	×	×	×	×
	G400	3.5	×	×	×	×	×	×
	G500	3.5	×	×	×	×	×	×
(1/5Strep,1P)-7	G100	2	×	×	×	×	×	×
	G200	3	×	×	×	×	×	×
	G300	3.5	×	×	×	×	×	×
	G400	6	×	×	×	×	×	×
	G500	6	×	×	1–5%	×	×	×
(1/5Strep,1P)-8	G100	2	×	×	×	×	×	×
	G200	2.5	×	×	×	×	×	×
	G300	3.5	×	×	×	×	×	×
	G400	3.5	×	×	×	×	×	×
	G500	4.5	×	×	×	×	×	×
(1/5Strep,10P)-1	G100	1.5	×	×	×	×	×	×
	G200	3	×	×	×	×	×	×
	G300	3	×	×	×	×	×	×
	G400	3	×	×	×	×	×	×
	G500	3.5	×	×	×	×	×	×
(1/5Strep,10P)-2	G100	2	×	×	×	×	×	×
	G200	3.5	×	×	×	×	×	×
	G300	2.5	×	×	×	×	×	×
	G400	2.5	×	×	×	×	×	×
	G500	4	×	×	×	×	×	×
(1/5Strep,10P)-3	G100	1.5	×	×	1–5%	×	×	×
	G200	3	1–5%	×	×	×	×	×
	G300	3	×	×	×	×	×	×
	G400	3.5	×	×	×	×	×	×
	G500	3.5	×	×	×	×	×	×
(1/5Strep,10P)-4	G100	1.5	×	×	1–5%	×	×	×
	G200	3	×	×	×	×	×	×
	G300	3	1%	×	×	×	×	×
	G400	3	×	×	×	×	×	×
	G500	9	×	×	×	×	×	×
(1/5Strep,10P)-5	G100	2	×	×	×	×	×	×
	G200	25	×	16%	5–10%	×	20%	20%
	G300	25	×	1–5%	×	×	20%	20%
	G400	25	×	×	×	×	100%	100%
	G500	25	×	×	×	×	100%	100%
(1/5Strep,10P)-6	G100	2	×	×	×	×	×	×
	G200	6	1%	1–5%	1–5%	×	< 1%	< 1%
	G300	9	×	5%	1–5%	×	1–5%	1–5%
	G400	8	×	×	×	×	×	×
	G500	8	×	×	×	×	×	×
(1/5Strep,10P)-7	G100	1.5	×	×	×	×	×	×
	G200	3.5	1%	×	5%	×	×	×
	G300	3.5	×	×	5–10%	×	×	×
	G400	15	×	×	10%	×	1%	1%
	G500	20	×	×	×	×	100%	100%

Lineage	Generation	MIC (×MIC_0)	*nuoG* (E10*)	*nuoG* (S548*)	*glnE* (A423V)	*sbmA* (E282*)	*rpsL* (R86S)	*dsbC* (V172E)
(1/5Strep,10P)-8	G100	2	×	×	1–5%	×	×	×
	G200	3.5	×	×	×	×	×	×
	G300	3.5	×	×	×	×	×	×
	G400	4	×	×	×	×	×	×
	G500	4.5	×	×	5%	×	×	×
(1/5Strep,100P)-1	G100	1.5	×	×	1–5%	×	×	×
	G200	2	×	×	×	×	×	×
	G300	3	×	×	×	×	×	×
	G400	3	×	×	×	×	×	×
	G500	3.5	×	×	×	×	×	×
(1/5Strep,100P)-2	G100	1.5	×	×	1–5%	×	×	×
	G200	3	1%	×	×	×	×	×
	G300	3	×	×	×	×	×	×
	G400	3.5	×	×	×	×	×	×
	G500	4	×	×	×	×	×	×
(1/5Strep,100P)-4	G100	2	×	×	×	×	×	×
	G200	3	×	×	×	×	×	×
	G300	3	×	5–10%	25%	×	×	×
	G400	3.5	×	×	×	×	×	×
	G500	9	×	×	×	×	1%	1%
(1/5Strep,100P)-8	G100	2	×	×	×	×	×	×
	G200	3	×	×	×	×	×	×
	G300	3.5	×	×	×	×	×	×
	G400	3	×	×	×	×	×	×
	G500	4	×	×	×	×	×	×
(0,100P)-1	G500	1	×	×	×	×	×	×
(0,100P)-2	G500	1	×	×	×	×	×	×
(0,100P)-3	G500	1.5	×	×	×	×	×	×
(0,100P)-4	G500	1.5	×	×	×	×	×	×
(0,100P)-5	G500	1.5	×	×	×	×	×	×
(0,100P)-6	G500	1.5	×	×	×	×	×	×
(0,100P)-7	G500	1.5	×	×	×	×	×	×
(0,100P)-8	G500	1.5	×	×	×	×	×	×
(1/2 Strep,0)-2	G200	15	×	×	×	×	×	×
(1/2 Strep,0)-4	G200	3	×	×	×	×	×	×
(1/2 Strep,0)-5	G200	6	×	×	×	×	×	×
(1/2 Strep,0)-6	G200	3	×	×	×	×	×	×
(1/2 Strep,0)-7	G200	10	×	×	×	×	×	×

[a]population MIC; [b]not detected; [c]semiquantification result of mutation frequency in the population by SNP assays.

Table S7 Strep resistance developed in *E. coli* populations exposed to Strep only at 1/2 MIC_0 (1/2Strep,0)

**Population**	**(1/2Strep,0) at 50G**	**(1/2Strep,0) at 100G**	**(1/2Strep,0) at 200G**
Rep-1	≥20× MIC_0	≥20× MIC_0	≥20× MIC_0
Rep-2	3× MIC_0	5× MIC_0	15× MIC_0
Rep-3	3× MIC_0	3× MIC_0	≥20× MIC_0
Rep-4	2× MIC_0	3× MIC_0	3× MIC_0
Rep-5	2× MIC_0	5× MIC_0	6× MIC_0
Rep-6	2× MIC_0	3× MIC_0	3× MIC_0
Rep-7	2× MIC_0	3× MIC_0	10× MIC_0
Rep-8	3× MIC_0	≥20× MIC_0	≥20× MIC_0

Table S8 The detection of *rpsL* and *dsbC* mutations in 10 randomly picked isolates from (1/5Strep,100P)-3 and (1/5Strep,10P)-5 populations using non-selective LB agar plates

Population	Frequency of *rpsL* and/or *dsbC* mutants	Genotypes in each mutant
(1/5Strep,100P)-3-G100	0/10	N.D.
(1/5Strep,100P)-3-G200	0/10	N.D.
(1/5Strep,100P)-3-G300	0/10	N.D.
(1/5Strep,100P)-3-G400	3/10	*rpsL* & *dsbC*
(1/5Strep,100P)-3-G500	9/10	*rpsL* & *dsbC*
(1/5Strep,10P)-5-G100	0/10	N.D.
(1/5Strep,10P)-5-G200	2/10	*rpsL* & *dsbC*
(1/5Strep,10P)-5-G300	2/10	*rpsL* & *dsbC*
(1/5Strep,10P)-5-G400	10/10	*rpsL* & *dsbC*
(1/5Strep,10P)-5-G500	10/10	*rpsL* & *dsbC*

N.D.: not detected

Table S9 Competition tests under different selection conditions

**Selection condition**	*nuoG*[1]+*glnE*+*sbmA*: wild type		*glnE* +*yaiW*: wild type		*nuoG*[2]: wild type		*rpsL*+*dsbC*+*rsmG*[1]: wild type	
	G0	G9	G0	G9	G0	G9	G0	G9
No stress	40%	25%	30%	15%	30%	15%	30%	17%
(0,100P)	40%	25%	30%	15%	30%	15%	30%	15%
(1/5Strep,0)	40%	45%	30%	40%	30%	35%	30%	60%
(1/5Strep,100P)	40%	50%	30%	40%	30%	36%	30%	60%

Table S10 MSC of resistant mutants from (1/5Strep, 100P) populations

Mutant genotype	MSC (of MIC_0)	MSC (absolute value μg/L)
nuoG[1] + *glnE* + *sbmA*	1/10 MIC_0	800
glnE + *yaiW*	1/10 ~ 1/20 MIC_0	400 ~ 800
nuoG[2]	1/20 MIC_0	400
rpsL + *dsbC* + *rsmG*[1]	1/40 MIC_0	200

Table S11 The change of *rpsL* mutant fraction in (1/2Strep,0)-1-G200 populations and (1/5Strep,100P)-5-G500 population after grown in LB medium without the corresponding selection pressure for 50 generations

Population	Fraction of *rpsL* mutants before the no-stress growth	Fraction of *rpsL* mutants after the no-stress growth
(1/2Strep,0)-1-G200	~100%	5%
(1/5Strep,100P)-5-G500	~100%	~100%

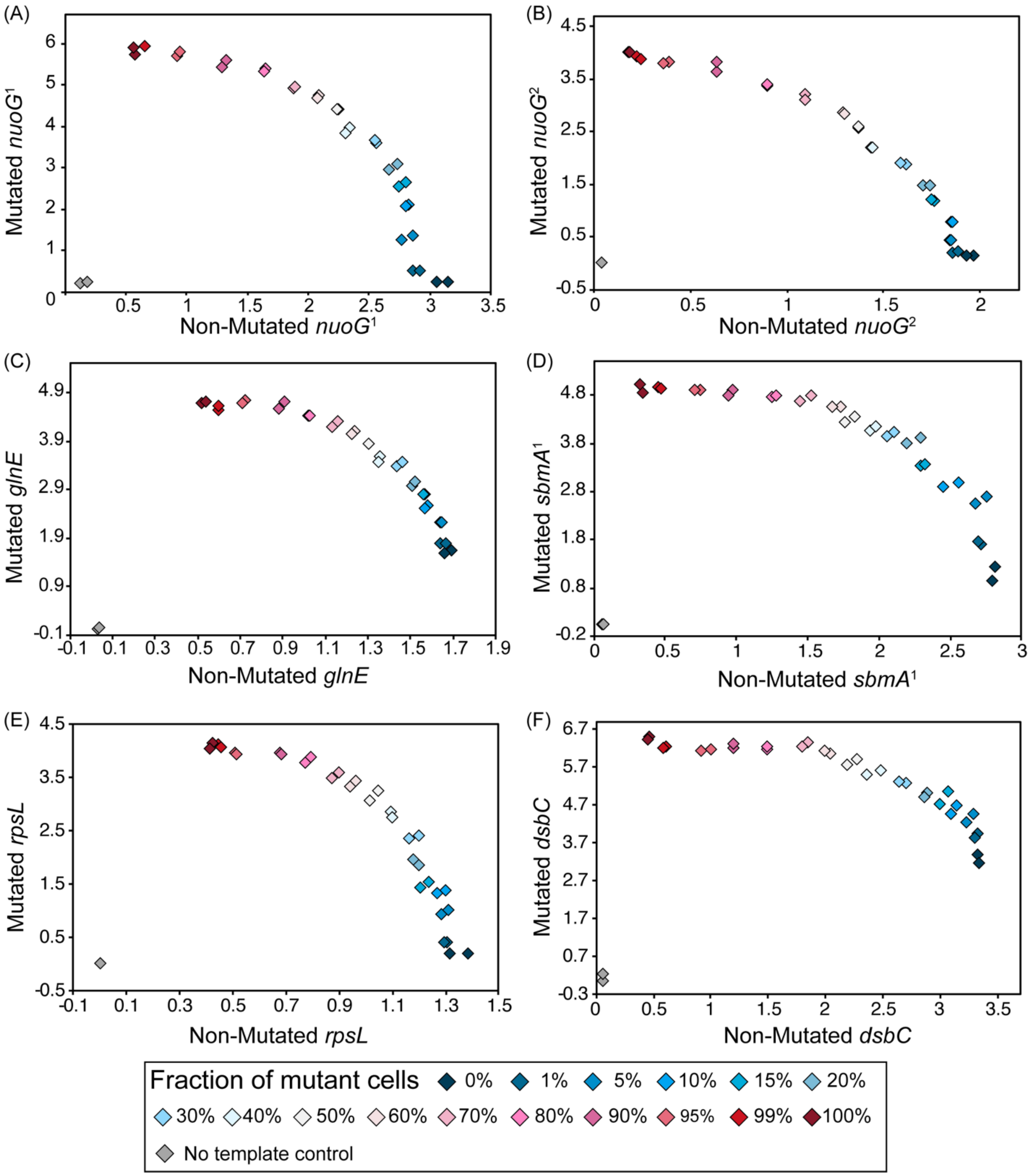

Fig. S1. Allelic discrimination plots obtained for *nuoG*[1] (Glu10*) (A), *nuoG*[2] (Ser548*) (B), *glnE* (Ala423Val) (C), *sbmA*[1] (Glu282*) (D), *rpsL* (Arg86Ser) (E), and *dsbC* (Val172Glu) (F) SNP mutations on standard mixtures at different fractions of mutant cells.

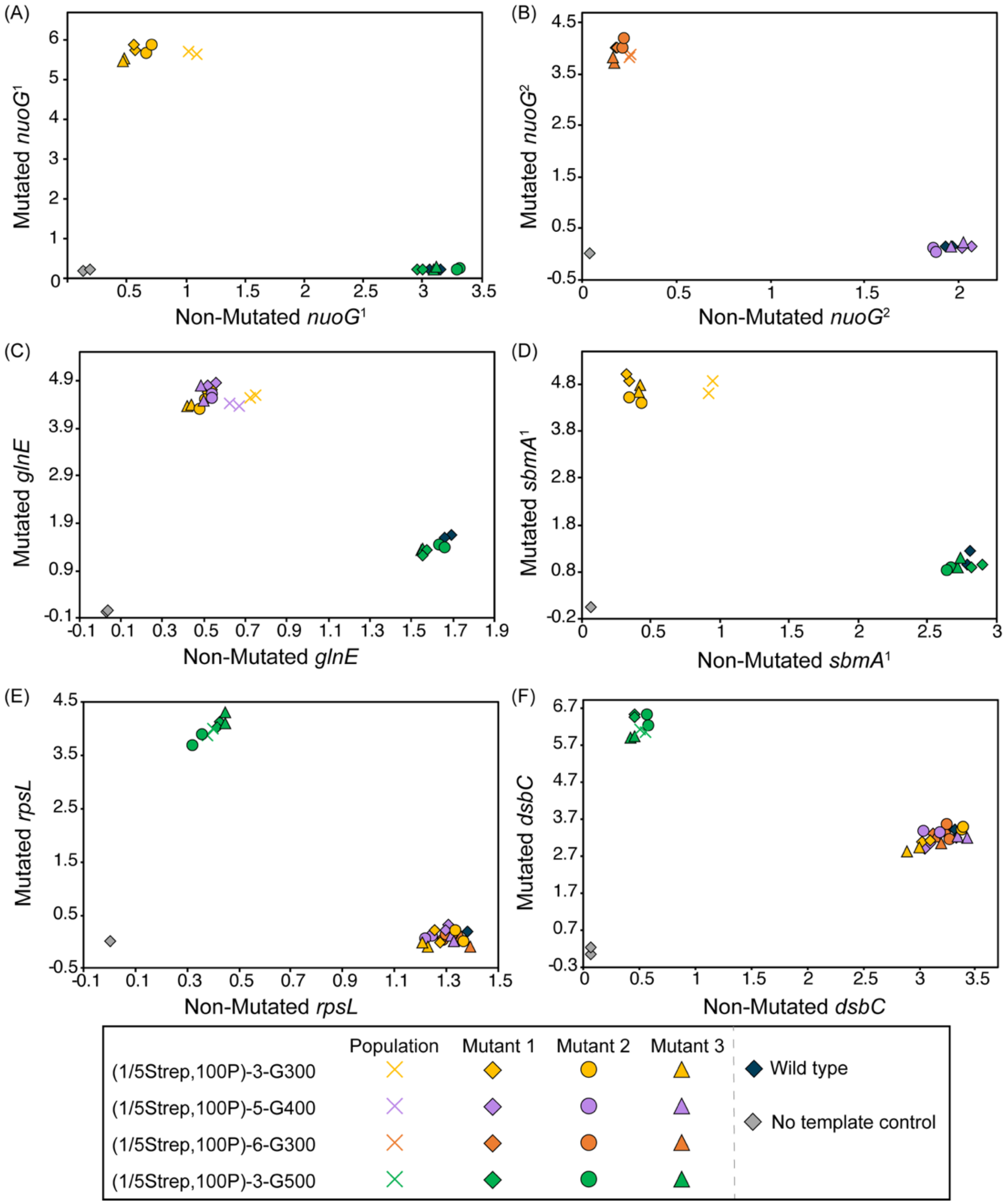

Fig. S2. Allelic discrimination plots obtained for *nuoG*[1] (Glu10*) (A), *nuoG*[2] (Ser548*) (B), *glnE* (Ala423Val) (C), *sbmA*[1] (Glu282*) (D), *rpsL* (Arg86Ser) (E), and *dsbC* (Val172Glu) (F) SNP mutations on isolated mutants and their originated populations.

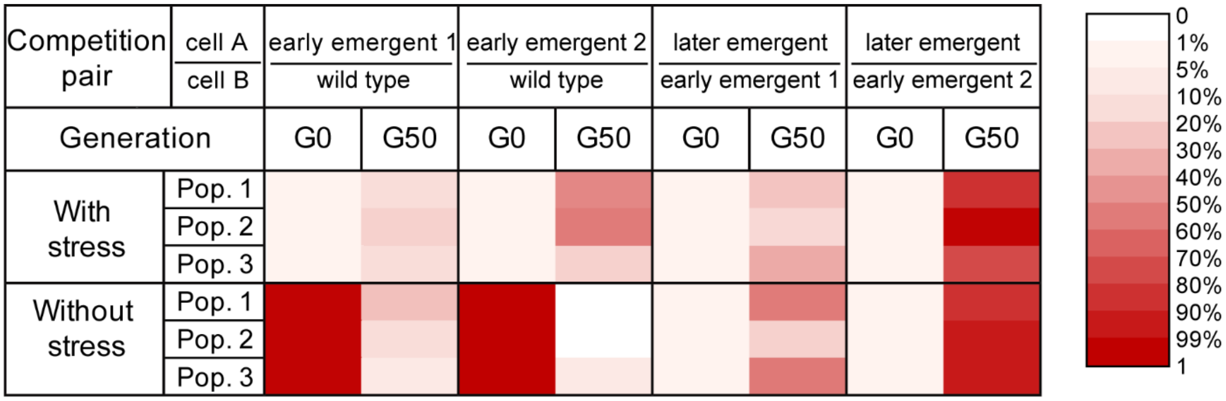

Fig. S3. Growth competition between the wild type, the two early emergent mutants with mild Strep resistance [early emergent 1: with *nuoG*^{2} (Ser548*) mutation; early emergent 2: with *glnE* (Ala423Val) and *yaiW* (Phe183Ile, Gln186Asp, His187fs) mutations], and the late emergent mutant with strong Strep resistance, carrying *rpsL*, *dsbC*, and *rsmG*^{1} (Trp150fs) mutations, in LB medium with and without the selection pressure (1/5Strep,100P) (Three parallel populations containing cell type A and B were performed with an initial fraction of cell A at 1% and 99%).
